# Establishment of a synthetic ECV model and its prognostic value in diabetes patients with acute myocardial infarction

**DOI:** 10.3389/fendo.2025.1534236

**Published:** 2025-06-25

**Authors:** Lei Chen, Bowen Qiu, Xinjia Du, Jiahua Liu, Yuan Lu, Wenliang Che, Wensu Chen

**Affiliations:** ^1^ Department of Cardiology, The Affiliated Hospital of Xuzhou Medical University, Xuzhou, China; ^2^ Department of Cardiology, Shanghai Tenth People’s Hospital, Tongji University School of Medicine, Shanghai, China

**Keywords:** cardiac magnetic resonance, synthetic extracellular volume, diabetes, acute myocardial infarction, major adverse cardiac events

## Abstract

**Background:**

Extracellular volume (ECV) is an important marker of myocardial fibrosis. However, the prognostic role of ECV in diabetes patients is unknown. In addition, synthetic ECV without blood sampling has not been reported in diabetes cohorts. This study investigated the establishment and validation of synthetic ECV and its prognostic value in type 2 diabetes mellitus (T2DM) patients with acute myocardial infarction (AMI).

**Methods:**

This single-center retrospective study included T2DM patients with AMI who completed cardiac magnetic resonance (CMR) during hospitalization. The patients were randomly divided into a derivation group and a validation group. MACE included all-cause death, recurrent MI, stroke, or heart failure. ECV in integral (Integral ECV), non-myocardial infarction region (NMI-ECV), and myocardial infarction region (MI-ECV) was obtained by CMR.

**Results:**

The study included 157 patients, with a median time from admission to CMR of 4 days. Bland-Altman and Pearson analysis showed good consistency and correlation between conventional ECV and synthetic ECV. Cox regression showed that Integral ECV (HR=1.07; 95%CI: 1.01 ~ 1.13, p = 0.023), MI-ECV (HR=1.03; 95%CI: 1.00 ~ 1.07, p = 0.024), and NMI-ECV (HR=1.07; 95%CI: 1.00 ~ 1.14, p = 0.039) were independently associated with MACE in different models. Kaplan-Meier analysis indicated that patients with a high synthetic ECV had a significantly higher MACE risk.

**Conclusions:**

Synthetic ECV is strongly consistent and correlated with conventional ECV in T2DM patients with AMI. Elevated synthetic ECV is an independent risk factor for MACE in T2DM patients with AMI.

## Introduction

Acute myocardial infarction (AMI) is one of the leading causes of death in the population worldwide, and type 2 diabetes mellitus (T2DM) is a major risk factor for AMI (1). Patients with diabetes have a higher risk of major adverse cardiovascular events (MACE) after AMI compared to patients without diabetes (2, 3). Although the pathophysiology of diabetes leading to cardiovascular disease is not fully understood, what can be confirmed is that myocardial fibrosis plays a key role (4). Myocardial fibrosis has been found to occur in diabetic patients in previous autopsy studies even in the absence of signs of ischemic heart disease (5, 6). Thus, myocardial fibrosis may play an important role in T2DM patients with AMI.

Histology remains the gold standard for myocardial fibrosis. However, endomyocardial biopsy is an invasive procedure that is neither reasonable nor feasible in the acute phase of AMI (7). Among the various noninvasive imaging modalities, cardiac magnetic resonance (CMR) has emerged as the most powerful tool for characterizing structural and functional changes in tissues. Extracellular volume (ECV) allows quantification of the extracellular matrix with good reproducibility and is termed “noninvasive” or “virtual biopsy” (8–10). As an alternative to myocardial fibrosis, ECV has been shown to independently predict MACE risk in a variety of diseases, including diabetes (11).

Conventional ECV measurements need to be combined with hematocrit (HCT), but HCT is highly individualized and is affected by several factors such as time of day and body position (12–14). To minimize the interference of variability, the Society for Cardiovascular Magnetic Resonance (SCMR) recommends blood collection within 24 hours of the CMR scan, which limits the routine clinical application of ECV measurement (15). In addition, conventional ECV is estimated from venous blood HCT, whereas ideally ECV calculations should be obtained from a left ventricular blood pool representing arterial blood (16). Indeed, a linear relationship between HCT and blood pool R1 (1/T1) has been extensively described (16–19). Based on this linear relationship, a method to determine synthetic ECV without blood sampling has been proposed. In these studies, good agreement was demonstrated between synthetic and conventional ECV (16, 20–22). However, the establishment and validation of synthetic ECV in diabetes patients have not been reported, and the prognostic value of different regional ECV in diabetes patients is unclear.

The main objectives of this study were as follows: First, to investigate the establishment and validation of synthetic ECV without blood sampling in T2DM patients with AMI; Second, to investigate the prognostic value of ECV from different regions in T2DM patients with AMI.

## Methods

### Study population

This retrospective study included T2DM patients with AMI at the Affiliated Hospital of Xuzhou Medical University from May 2019 to June 2024. Inclusion criteria: 1. Underwent CMR with T1 mapping sequences during hospitalization; 2. Underwent coronary angiography (CAG) and successful revascularization therapy (TIMI ≥ 2). Exclusion criteria: 1. Without HCT within 24 hours of the CMR examination; 2. Poor image quality; 3. History of myocardial infarction; 4. Malignancy, or inflammatory disease; 5. Severe renal insufficiency; 6.Blood disease. The Institutional Review Board (IRB) approved the study protocol (XYFY2023-KL199-01). Considering this was a retrospective study with no risk to patients, the informed consent was waived by the IRB. The patients were randomly divided into a derivation group (n = 80) and a validation group (n = 77). The clinical data, relevant laboratory indexes, and medications were obtained from the patient’s clinical records. Peak values of high-sensitivity troponin T (hs-TnT), high-sensitivity C-reactive protein (hs-CRP), and N-terminal pro-B-type natriuretic peptide (NT-proBNP) during hospitalization were collected. Infarct-related arteries (IRA) were recorded based on CAG. Considering the interference of stress glucose elevation, only patients diagnosed with T2DM before the current AMI were included in this study (**Supplementary Figure 1**).

### Cardiac MRI protocol and cardiac MRI-related parameters

The median time to CMR completion was 4 (3.5, 6) days after hospitalization. The detailed parameters of CMR have been described in our earlier publications (23, 24). CMR assessments were conducted using 3.0 T imaging systems (Ingenia, Philips, The Netherlands). A balanced turbo field echo (BTFE) sequence was implemented. Scan parameters: slice thickness = 7 mm, no interlayer gap; echo time (TE) = 1.47 ms, repetition time (TR) = 2.94 ms; flip angle = 60°, field of view (FOV) = 300 × 300 mm, matrix = 280 × 240 mm and voxel size = 1.22 × 1.22 × 8.0 mm^3^. Following the administration of a gadolinium-based contrast agent (0.1 mmol/kg), short-axis images encompassing the left ventricle (LV) were obtained with 3 to 5 slices, and T1-mapping (MOLLI) was performed 10 to 15 minutes both before and after the application of the contrast medium. The CVI42 (cvi42^®^ version 5.13.5, Circle Cardiovascular Imaging, Canada) was used for image analysis. The analysis of CMR images was carried out independently by two skilled physicians who did not know this study. End-diastolic volume (EDV), end-systolic volume (ESV), and left ventricular ejection fraction (LVEF) values were automatically derived and adjusted for body surface area (BSA). CMR feature tracking was used to measure the global longitudinal strain (GLS). Microvascular obstruction (MVO) and late gadolinium enhancement (LGE) were adjusted for the total left ventricular myocardial mass. The endocardial, epicardial, and blood pool were identified on a short-axis view to evaluate the T1 relaxation time. Average relaxation times (≥1 cm²) for two regions of interest (ROI) were recorded at both the myocardial infarction region and the non-infarction region. The limbic area and papillary muscles were meticulously avoided. The T1 value concerning the blood pool was derived from the left ventricle. Conventional ECV was computed using previously established equations: ECV = (1-HCT) × (1/Myocardial enhanced T1 -1/Myocardial native T1)/(1/Blood pool enhanced T1 -1/Blood pool native T1). ECV in integral (Integral ECV), non-myocardial infarction region (NMI-ECV), and myocardial infarction region (MI-ECV) was obtained (**Supplementary Figure 2**).

### Calculation of synthetic ECV

Within the derivation group, an analysis was conducted on the reciprocal longitudinal relaxation time of blood (R1 = 1/T1) and HCT to explore their linear correlation, resulting in a formula for synthetic HCT. In the validation group, the synthetic ECV was then calculated by applying the synthetic HCT values. The synthetic ECV was verified by the analysis with conventional ECV in the validation group. In all patients, synthetic ECV from different regions was calculated to investigate the prognostic value in patients with AMI combined with diabetes.

### Clinical outcomes and follow-up

Patients were followed up from their discharge via outpatient appointments and/or telephone communications utilizing a standardized questionnaire. In cases where the patient was unreachable, pertinent information was obtained from the patient’s family members or healthcare provider. The main follow-up endpoint was MACE, which included all-cause mortality, recurrent myocardial infarction (MI), stroke, or heart failure. The diagnosis of recurrent MI and heart failure was made following the latest guidelines from the European Society of Cardiology (ESC) (25, 26). Stroke was characterized as neurological impairment and cerebrovascular damage resulting from either cerebral ischemia or hemorrhage (27). Any patients who could not be followed up were confirmed deceased through the local official household registry.

### Statistical analysis

Statistical analysis was performed using SPSS 26.0 (IBM, Chicago, USA). To evaluate the normality of the data, the Kolmogorov-Smirnov test was used. Continuous variables that followed a normal distribution were represented as mean ± standard deviation (SD), with subsequent analysis performed using Student’s t-test. Continuous variables that did not conform to normal distribution were summarized as median (Q25, Q75), and analyzed using a nonparametric test. Categorical variables were depicted in terms of frequencies and percentages, and analyzed through the chi-square test (count >5) or Fisher test (count ≤5). For the derivation group, linear regression analysis was conducted to derive the synthetic HCT formula. In the validation group, a Bland-Altman analysis was executed to evaluate the consistency between synthetic and conventional ECV. The Pearson correlation coefficient was calculated to assess relationships among continuous variable gates. To examine the association of ECV with MACE, Cox regression models were utilized. Variables associated with MACE (p < 0.1) in univariate analysis were incorporated into the multivariate model using a stepwise forward approach. To eliminate collinear interference, Integral ECV, NMI-ECV, and MI-ECV were analyzed in separate multivariate Cox regression models. Receiver operating characteristic (ROC) curve was applied to evaluate the capability of ECV in identifying MACE. Based on the cut-off values derived from the ROC analysis, all patients were stratified into two groups for Kaplan-Meier curve analysis. All P values are from two-sided tests and results were considered statistically significant at p < 0.05.

## Results

### Establishment of an ECV model without blood sampling

The derivation group was used to calculate the linear regression equation of synthetic HCT and ECV. There were no statistical differences in baseline characteristics between the derivation and validation groups (**Supplementary Table 1**). In the derivation group, R1 and conventional HCT had a linear correlation (R^2 = 0.29, p < 0.001). Through linear regression analysis, R1 was used to derive the formula to estimate synthetic HCT: HCT=697.95* (1/Blood pool T1) + 0.03207 (**Supplementary Figure 3**).

### Validation of an ECV model without blood sampling

**Figure 1:**
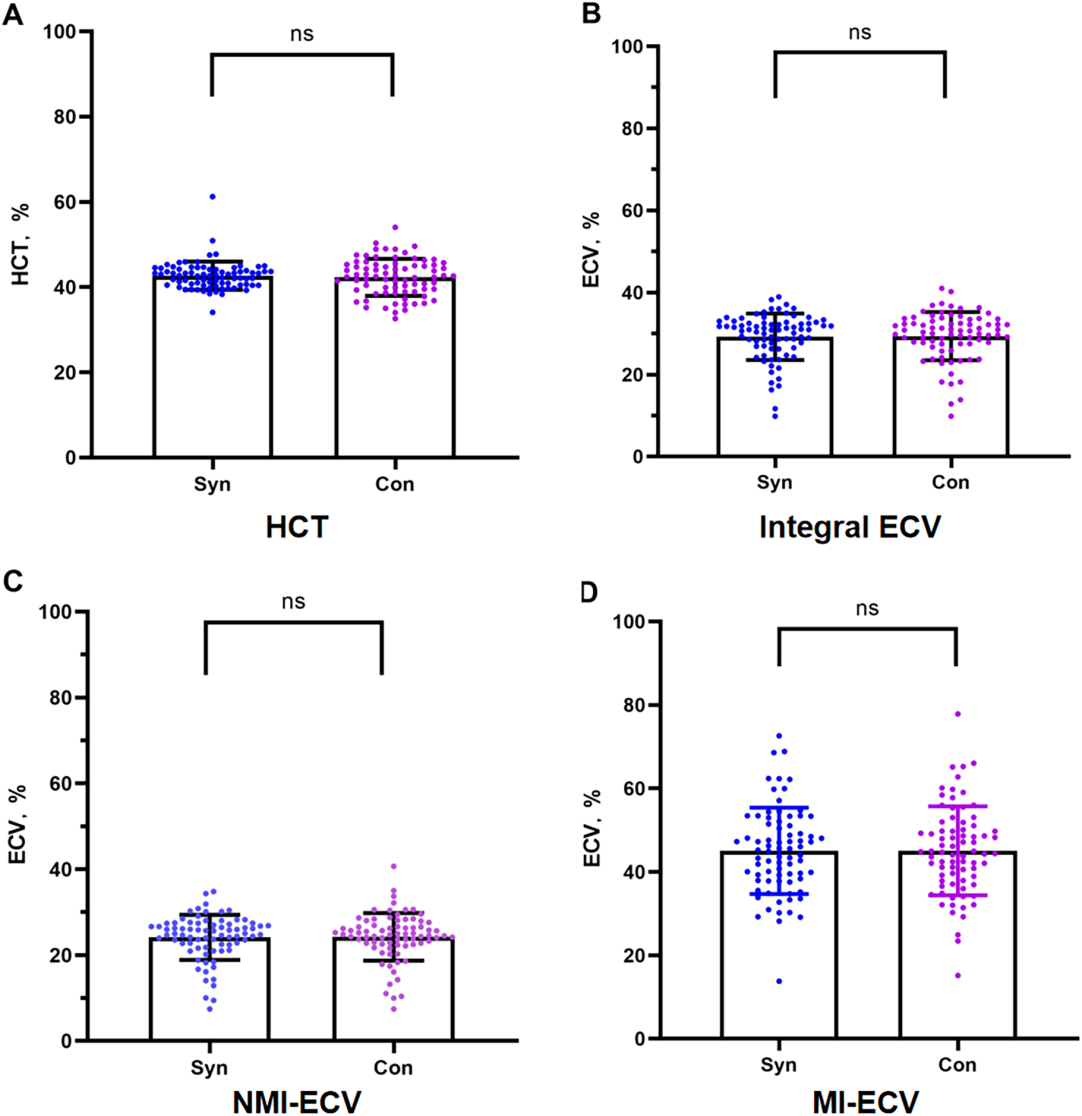
Comparison between conventional ECV and synthetic ECV. **(A)** Comparison between conventional HCT and synthetic HCT; **(B)** Comparison between conventional ECV and synthetic ECV in the integral myocardium; **(C)** Comparison between conventional NMI-ECV and synthetic NMI-ECV in NMI; **(D)** Comparison between conventional ECV and synthetic ECV at MI. NMI, non-myocardial infarction regions; MI, myocardial infarction regions; ECV, extracellular volume; HCT, hematocrit.

**Figure 2:**
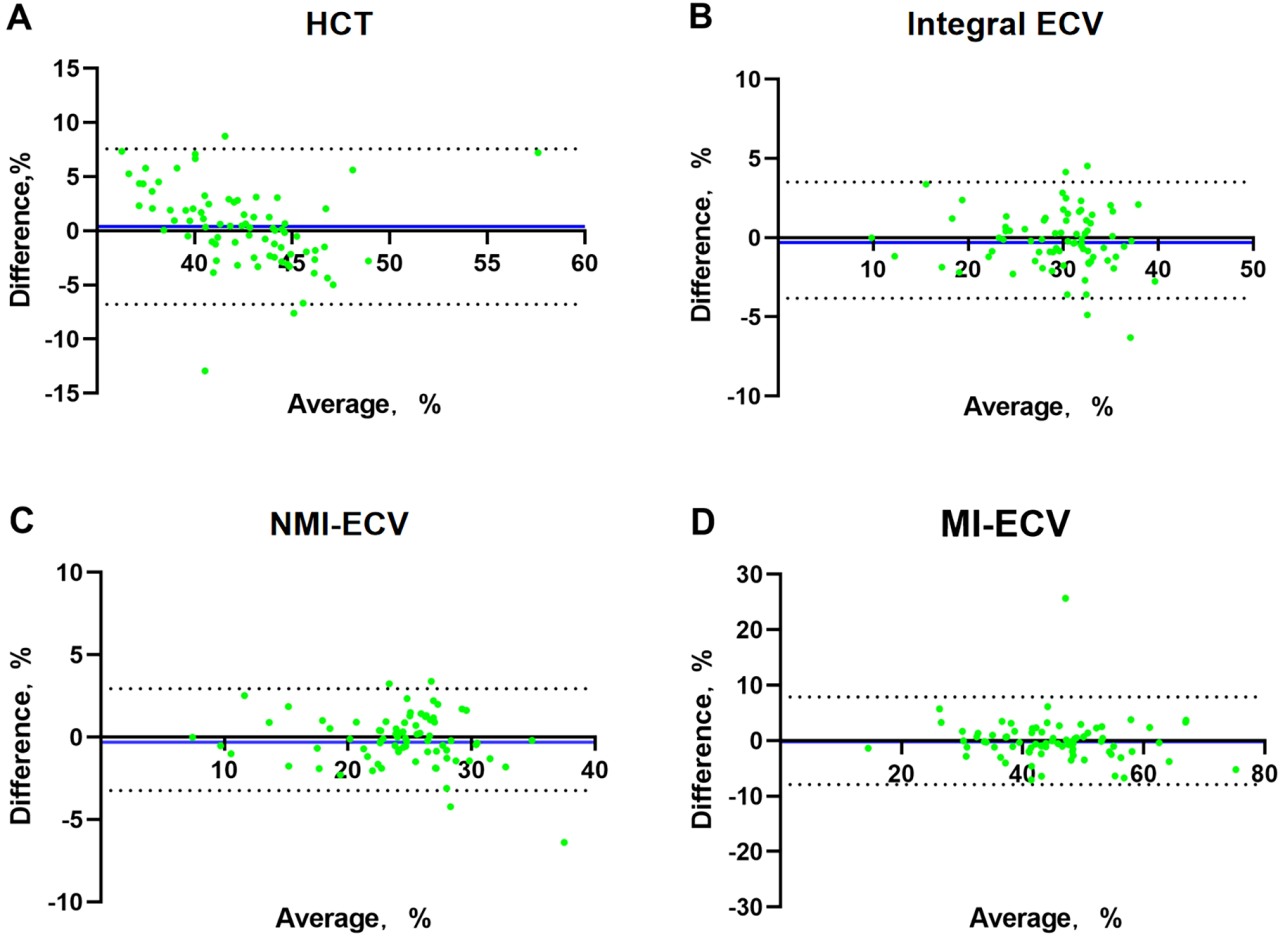
Bland-Altman analysis between conventional ECV and synthetic ECV. **(A)** Bland-Altman analysis between conventional HCT and synthetic HCT; **(B)** Bland-Altman analysis between conventional ECV and synthetic ECV in the integral myocardium; **(C)** Bland-Altman analysis between conventional ECV and synthetic ECV in NMI; **(D)** Bland-Altman analysis between conventional ECV and synthetic ECV in MI. The X-axis represents the mean value, The Y-axis represents Bias, The blue line is Bias. NMI, non-myocardial infarction regions; MI, myocardial infarction regions; ECV, extracellular volume; HCT, hematocrit.

**Figure 3:**
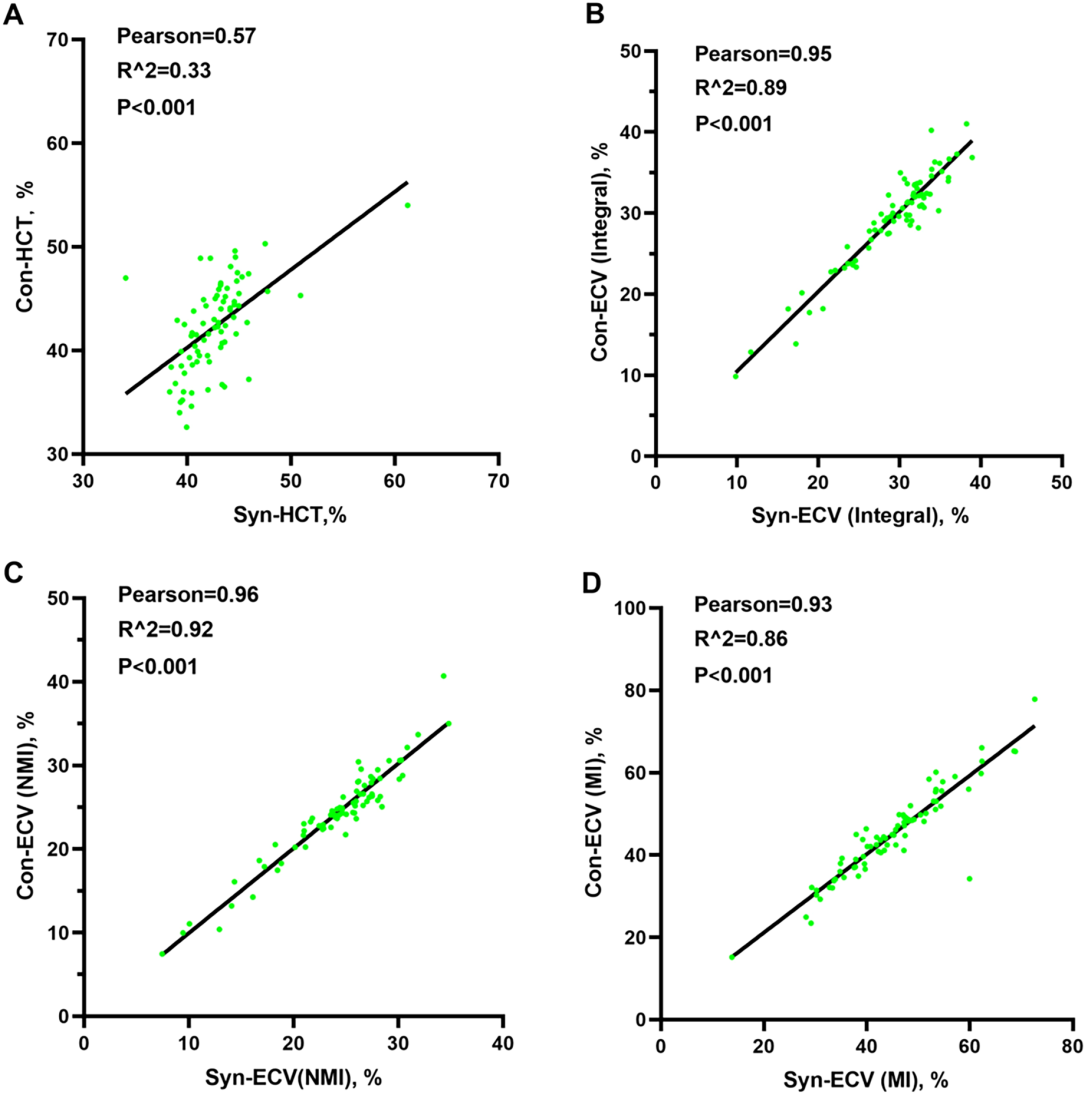
Pearson analysis between conventional ECV and synthetic ECV. **(A)** Pearson analysis between conventional HCT and synthetic HCT; **(B)** Pearson analysis between conventional ECV and synthetic ECV in the integral myocardium; **(C)** Pearson analysis between conventional ECV and synthetic ECV in NMI; **(D)** Pearson analysis between conventional ECV and synthetic ECV in MI. NMI, non-myocardial infarction region; MI, myocardial infarction regions; ECV, extracellular volume; HCT, hematocrit.

In the validation group, the formula was used to calculate the synthetic HCT. Then, the synthetic HCT was used to calculate synthetic ECV. In the validation group, there was no statistical difference between conventional HCT and synthetic HCT. Also, there was no statistical difference between conventional ECV and synthetic ECV from different regions (**Figure 1**; **Table 1**). Bland-Altman analysis showed good consistency between the conventional HCT and the synthetic HCT (Bias = 0.38). The synthetic ECV and conventional ECV in Integral, NMI, and MI also showed high consistency in the verification group (Bias = -0.16, -0.15, and -0.01, respectively) (**Figure 2**). In addition, the conventional HCT and the synthetic HCT showed a linear correlation (R^2 = 0.33, p<0.001); the synthetic ECV and conventional ECV in Integral, NMI, and MI also showed a good linear correlation (R^2 = 0.89, p<0.001; R^2 = 0.92, p<0.001; R^2 = 0.86, p<0.001; respectively) (**Figure 3**).

**Table 1:** Comparison of Pearson correlation coefficients of ECV.

Validation (n=77)	Conventional	Synthetic	P
HCT, %	42.02 ± 4.20	42.66 ± 3.32	0.294
Integral ECV, %	29.52 ± 6.10	29.21 ± 5.67	0.743
NMI-ECV, %	24.42 ± 5.93	24.11 ± 5.24	0.731
MI-ECV, %	45.28 ± 10.70	45.05 ± 10.36	0.893

HCT, haematocrit; ECV, extracellular volume; NMI, non-myocardial myocardial infarction; MI, myocardial infarction.

### Baseline characteristics of patients

A total of 157 AMI patients were enrolled in this study, including 102 patients with STEMI. There were 41 (26.1%) patients with MACE after a median time of 26.6 (17.1, 38.1) months of follow-up (**Supplementary Table 2**). Compared with No MACE group, patients with MACE had higher age, hs-TnT, NT-proBNP, LGE%, and MVO%, lower LVEF and GLS. In addition, the MI-ECV (50.52 ± 10.49% vs. 45.37 ± 10.24%, p = 0.007), NMI-ECV (26.06 ± 5.91% vs. 23.82 ± 5.18%, p = 0.023), and Integral ECV (32.73 ± 5.85% vs. 29.42 ± 6.25%, p = 0.003) in the MACE group were significantly higher than those in the No MACE group (**Table 2**).

**Table 2:** Patient characteristics.

Variables	No MACE (n = 116)	MACE (n = 41)	P
Age, years	56.84 ± 11.26	61.41 ± 11.54	0.028
Female, n (%)	22 (19.0)	5 (12.2)	0.323
BMI, kg/m^2^	25.96 ± 3.38	25.5 ± 2.71	0.426
Smoker, n (%)	54 (46.6)	18 (43.9)	0.770
Hypertension, n (%)	53 (45.7)	16 (39.0)	0.460
Stroke, n (%)	17 (14.7)	7 (17.1)	0.712
STEMI, n (%)	72 (62.1)	30 (73.2)	0.200
SBP, mmHg	130.8 ± 19.71	125.68 ± 16.88	0.141
DBP, mmHg	79.8 ± 13.39	79.15 ± 13.85	0.790
Heart rate, bpm	80.39 ± 14.19	79.71 ± 16.12	0.799
Total cholesterol, mmol/L	4.27 ± 1.08	4.52 ± 0.82	0.183
Triglycerides, mmol/L	1.94 ± 1.55	1.84 ± 1.24	0.713
HDL cholesterol, mmol/L	0.96 ± 0.24	0.94 ± 0.2	0.666
LDL cholesterol, mmol/L	2.63 ± 0.94	2.91 ± 0.83	0.091
HbA1c, %	6.58 ± 1.25	6.68 ± 0.84	0.630
FBG, mmol/L	8.85 ± 3.49	9.09 ± 3.08	0.698
eGFR, mL/min/1.73 m^2^	109.81 ± 16.69	106.31 ± 16.65	0.249
hs-CRP, mg/L	13.75 (4.23, 47)	17.4 (7.25, 53.25)	0.342
hs-TnT, ng/L	2392.5 (773.4, 5072.8)	4942.0 (2480.5, 8388.5)	0.002
NT-proBNP, pg/mL	950.0 (545.0, 1932.3)	2155.0 (784.5, 3250.5)	0.003
Antiplatelet, n (%)	115 (99.1)	41 (100.0)	1.000
Statins, n (%)	112 (96.6)	38 (92.7)	0.326
Dapagliflozin, n (%)	84 (72.4)	31 (75.6)	0.691
Insulin, n (%)	42 (36.2)	18 (43.9)	0.383
ACEI/ARB, n (%)	76 (65.5)	25 (61.0)	0.602
β-Blockers, n (%)	101 (87.1)	34 (82.9)	0.511
Spironolactone, n (%)	12 (10.3)	6 (14.6)	0.469
Killip class, n (%)			0.768
I	106 (91.4)	36 (87.8)	
II	6 (5.2)	3 (7.3)	
III	1 (0.9)	0 (0.0)	
IV	3 (2.6)	2 (4.9)	
IRA, n (%)			
LCX	26 (22.4)	10 (24.4)	0.796
LAD	51 (44.0)	14 (34.1)	0.273
RCA	38 (32.8)	15 (36.6)	0.656
Others	1 (0.9)	2 (4.9)	0.167
Integral ECV, %	29.42 ± 6.25	32.73 ± 5.85	0.003
NMI-ECV, %	23.82 ± 5.18	26.06 ± 5.91	0.023
MI-ECV, %	45.37 ± 10.24	50.52 ± 10.49	0.007
GLS, %	13.79 ± 4.22	10.97 ± 4.27	<0.001
LVEF, %	46.71 ± 10.56	42.04 ± 11.01	0.017
LV-EDVi, mL/m^2^	77.49 ± 17.83	78.27 ± 24.24	0.827
LV-ESVi, mL/m^2^	43.73 ± 15.97	48.18 ± 20.53	0.158
MVO, n (%)	62 (53.4)	27 (65.9)	0.168
MVO%	0.21 (0, 3.19)	2.82 (0, 9.70)	0.007
LGE%	22.20 (15.25, 30.77)	31.34 (18.50, 42.20)	0.013

BMI, body mass index; STEMI, ST-segment elevation myocardial infarction; GFR, glomerular filtration rate; ECV, extracellular volume; LVEF, left ventricular ejection fraction; GLS, global longitudinal strain; LV, left ventricular; EDVi, end-diastolic volume index; LGE, late gadolinium enhanced; MVO, microvascular obstruction; ESVi, end-systolic volume index; SBP, systolic blood pressure; DBP, diastolic blood pressure; LAD, left atrial diameter; LCX, left circumﬂex artery; RCA, right coronary artery; ACEI, angiotensin-converting-enzyme inhibitor; IRA, infarct-related artery; ARB, angiotensin II receptor blocker; HDL-C, high-density leptin cholesterol; LDL-C, low-density leptin cholesterol; Others, left main coronary artery and intermediate branch; hs-CRP, high sensitivity C-reactive protein; hs-TnT, high sensitivity troponin T; NT-proBNP, N-terminal pro-B-type natriuretic peptide; MI, myocardial infarction; NMI, non-myocardial infarction.

### Relationship between synthetic ECV and MACE

**Figure 4:**
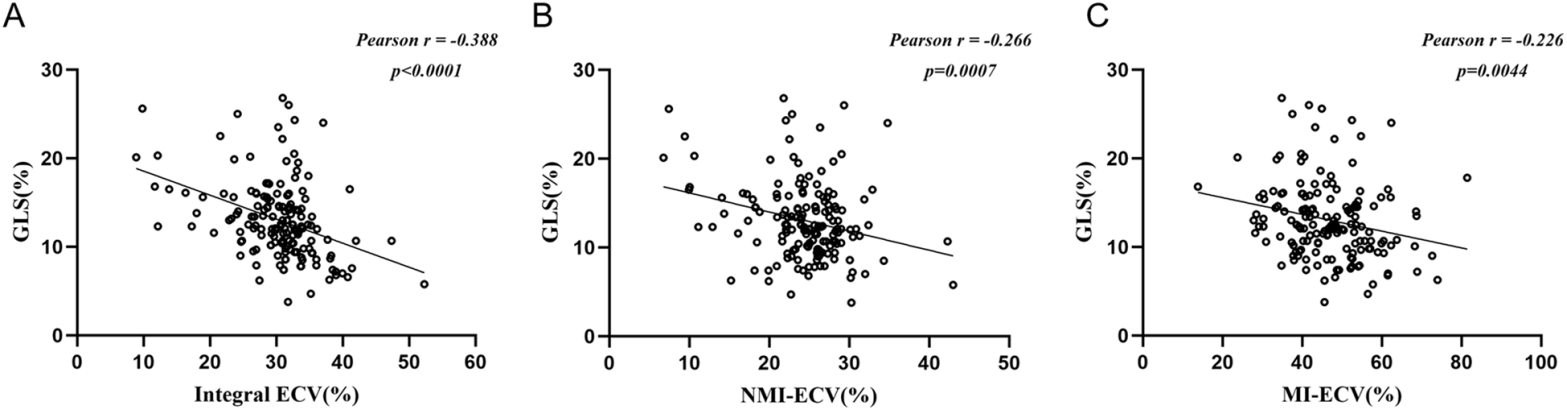
Pearson analysis between synthetic ECV and GLS. **(A)** Pearson analysis between GLS and synthetic ECV in the integral myocardium; **(b)** Pearson analysis between GLS and synthetic ECV in NMI; **(C)** Pearson analysis between GLS and synthetic ECV in MI. GLS, global longitudinal strain; NMI, non-myocardial infarction regions; MI, myocardial infarction regions; ECV, extracellular volume.

Univariate COX regression analysis identified hs-TnT, NT-proBNP, MI-ECV, NMI-ECV, Integral ECV, GLS, LVEF, LGE%, and MVO% associated with MACE. Integral ECV, NMI-ECV, and MI-ECV were included in model 1, model 2, and model 3, respectively. After adjusting for confounding factors, it was found that hs-TnT, GLS, MVO%, Integral ECV (HR=1.07; 95%CI: 1.01 ~ 1.13, p = 0.023), and NMI-ECV (HR=1.07; 95%CI: 1.00 ~ 1.14, p = 0.039) were independently associated with MACE in model 1 and model 2. In model 3, it was found that hs-TnT, GLS, and MI-ECV (HR=1.03; 95%CI: 1.00 ~ 1.07, p = 0.024) were independently associated with MACE (**Table 3**, **4**). GLS was moderately correlated with Integral ECV (r = -0.388, p < 0.001), weakly correlated with NMI-ECV (r = -0.266, p < 0.001) and NMI-ECV (r = -0.226, p = 0.004) (**Figure 4**).

**Table 3:** Association of patient characteristics With MACE: univariate and multivariate analysis.

Variables	Univariate		Multivariate	
			Integral ECV (Model 1)	
	HR (95%CI)	P	HR (95%CI)	P
Age, years	1.02 (0.99 ~ 1.05)	0.173		
Female, n (%)	0.65 (0.25 ~ 1.65)	0.360		
BMI, kg/m^2^	0.97 (0.88 ~ 1.07)	0.524		
Current smoker, n (%)	0.86 (0.46 ~ 1.62)	0.644		
Hypertension, n (%)	0.76 (0.41 ~ 1.44)	0.405		
Stroke, n (%)	0.49 (0.15 ~ 1.59)	0.235		
STEMI, n (%)	1.67 (0.76 ~ 3.66)	0.203		
SBP, mmHg	0.99 (0.97 ~ 1.00)	0.172		
DBP, mmHg	1.00 (0.97 ~ 1.02)	0.714		
Heart rate, bpm	1.00 (0.98 ~ 1.02)	0.764		
Total cholesterol, mmol/L	1.18 (0.87 ~ 1.61)	0.294		
Triglycerides, mmol/L	1.00 (0.78 ~ 1.27)	0.983		
HDL cholesterol, mmol/L	0.61 (0.15 ~ 2.42)	0.478		
LDL cholesterol, mmol/L	1.26 (0.90 ~ 1.78)	0.179		
HbA1c, %	1.04 (0.83 ~ 1.32)	0.726		
FBG, mmol/L	1.02 (0.93 ~ 1.11)	0.699		
eGFR, mL/min/1.73 m^2^	0.99 (0.98 ~ 1.01)	0.524		
hs-CRP, mg/L	1.00 (0.99 ~ 1.01)	0.989		
hs-TnT, ng/L	1.62 (1.18 ~ 2.23)	0.003	1.57 (1.14 ~ 2.16)	0.006
NT-proBNP, pg/mL	1.41 (1.04 ~ 1.91)	0.028		
Statins, n (%)	0.62 (0.19 ~ 2.01)	0.427		
Dapagliflozin, n (%)	1.07 (0.52 ~ 2.20)	0.846		
Insulin, n (%)	1.38 (0.67 ~ 2.84)	0.384		
ACEI/ARB, n (%)	0.92 (0.48 ~ 1.75)	0.800		
β-Blockers, n (%)	0.98 (0.41 ~ 2.33)	0.959		
Spironolactone, n (%)	1.37 (0.58 ~ 3.27)	0.474		
Killip ≥2, n (%)	1.47 (0.47 ~ 4.60)	0.505		
IRA-LCX, n (%)	0.95 (0.45 ~ 2.00)	0.898		
IRA-LAD, n (%)	0.73 (0.38 ~ 1.41)	0.351		
IRA-RCA, n (%)	1.23 (0.65 ~ 2.34)	0.525		
Integral ECV, %	1.11 (1.05 ~ 1.18)	<0.001	1.07 (1.01 ~ 1.13)	0.023
NMI-ECV, %	1.10 (1.03 ~ 1.18)	0.007	Not in model	
MI-ECV, %	1.04 (1.02 ~ 1.07)	0.002	Not in model	
GLS, %	0.84 (0.77 ~ 0.92)	<0.001	0.90 (0.81 ~ 0.99)	0.033
LVEF, %	0.97 (0.94 ~ 0.99)	0.025		
LV-EDVi, mL/m^2^	1.00 (0.99 ~ 1.02)	0.662		
LV-ESVi, mL/m^2^	1.01 (1.00 ~ 1.03)	0.137		
MVO, n (%)	1.33 (0.69 ~ 2.55)	0.390		
MVO%	1.08 (1.03 ~ 1.13)	0.002	1.06 (1.01 ~ 1.11)	0.023
LGE%	1.02 (1.01 ~ 1.04)	0.024		

BMI, body mass index; GFR, glomerular filtration rate; STEMI, ST-segment elevation myocardial infarction; ECV, extracellular volume; LVEF, left ventricular ejection fraction; GLS, global longitudinal strain; LV, left ventricular; EDVi, end-diastolic volume index; LGE, late gadolinium enhanced; MVO, microvascular obstruction; ESVi, end-systolic volume index; SBP, systolic blood pressure; DBP, diastolic blood pressure; LAD, left atrial diameter; LCX, left circumﬂex artery; RCA, right coronary artery; ACEI, angiotensin-converting-enzyme inhibitor; IRA, infarct-related artery; ARB, angiotensin II receptor blocker; HDL-C, high-density leptin cholesterol; LDL-C, low-density leptin cholesterol; Others, left main coronary artery and intermediate branch; hs-CRP, high sensitivity C-reactive protein; hs-TnT, high sensitivity troponin T; NT-proBNP, N-terminal pro-B-type natriuretic peptide; MI, myocardial infarction; NMI, non-myocardial infarction.

**Table 4:** Association of patient characteristics with MACE: multivariate analysis.

Variables	NMI-ECV (Model 2)		MI-ECV (Model 3)	
	HR (95%CI)	P	HR (95%CI)	P
hs-TnT, ng/L	1.57 (1.14 ~ 2.15)	0.006	1.56 (1.13 ~ 2.15)	0.007
NT-proBNP, pg/mL				
Integral ECV, %	Not in model		Not in model	
NMI-ECV, %	1.07 (1.00 ~ 1.14)	0.039	Not in model	
MI-ECV, %	Not in model		1.03 (1.00 ~ 1.07)	0.024
GLS%	0.88 (0.80 ~ 0.97)	0.009	0.86 (0.78 ~ 0.94)	0.001
LVEF, %				
MVO%	1.07 (1.01 ~ 1.12)	0.013		
LGE%				

ECV, extracellular volume; LVEF, left ventricular ejection fraction; GLS, global longitudinal strain; LGE, late gadolinium enhanced; MVO, microvascular obstruction; hs-TnT, high sensitivity troponin T; NT-proBNP, N-terminal pro-B-type natriuretic peptide; MI, myocardial infarction; NMI, non-myocardial infarction.

### Value of synthetic ECV for predicting MACE

**Figure 5:**
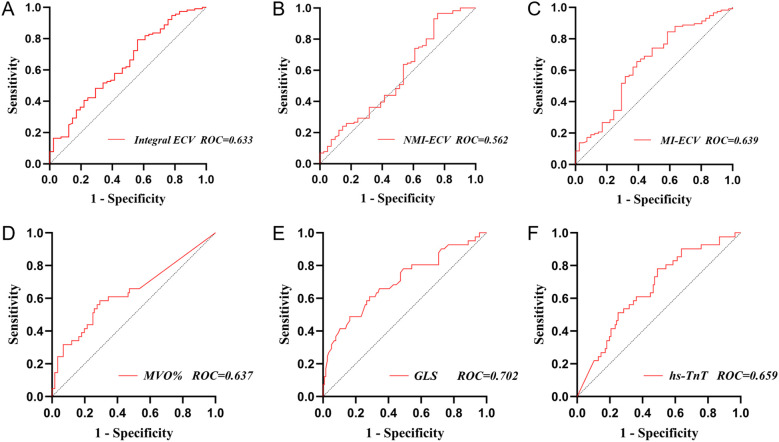
Receiver operating characteristic analysis of synthetic ECV for MACE. **(A)** ROC analysis of synthetic Integral ECV for MACE; **(B)** ROC analysis of synthetic NMI-ECV for MACE; **(C)** ROC analysis of synthetic MI-ECV for MACE; **(D)** ROC analysis of MVO for MACE; **(E)** ROC analysis of GLS for MACE; **(F)** ROC analysis of hs-TnT for MACE. GLS, global longitudinal strain; NMI, non-myocardial infarction regions; MI, myocardial infarction regions; ECV, extracellular volume; MVO, microvascular obstruction; hs-TnT, high sensitivity troponin T; MACE, major adverse cardiovascular events; ROC, receiver operating characteristic analysis.

**Figure 6:**
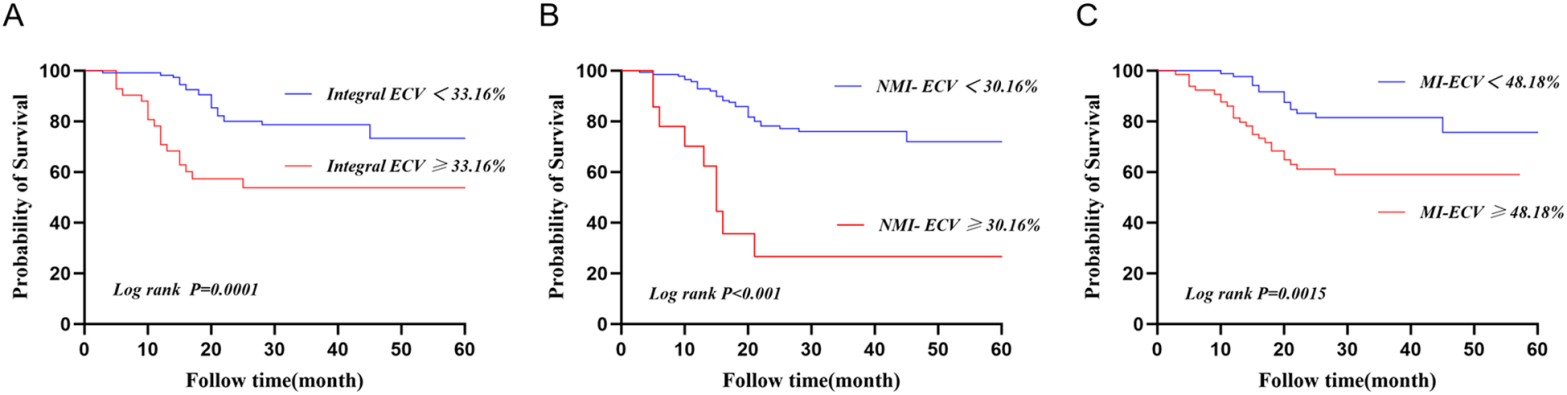
Kaplan-Meier curve for patients based on the cut-off values of synthetic ECV. **(A)** Kaplan-Meier curve of synthetic Integral ECV for MACE; **(B)** KaplanMeier curve of synthebtic NMI-ECV for MACE; **(C)** Kaplan-Meier curve of synthetic MI-ECV for MACE. ECV, extracellular volume; MACE, major adverse cardiovascular events.

ROC was used to analyze the predictive value of synthetic ECV for MACE. The results showed that the area under the curve (AUC) of MI-ECV for MACE was 0.639 (95% CI 0.537 ~ 0.741, p = 0.008) with a cut-off value of 48.18%, the AUC of Integral ECV for MACE was 0.633 (95% CI 0.533 ~ 0.733, p = 0.011) with a cut-off value of 33.16%, and the AUC of NMI-ECV for MACE was 0.562 (95% CI 0.455 ~ 0.670, p = 0.236) with a cut-off value of 30.16% (**Figure 5**; **Table 5**). Based on the cut-off value of synthetic ECV, all patients were stratified into two groups for Kaplan-Meier curve analysis. Kaplan-Meier curve showed that compared with the AMI patients with low synthetic ECV, the patients with high synthetic ECV had a significantly higher long-term risk of MACE (both log-rank P < 0.05) (**Figure 6**).

**Table 5:** ROC of parameters for MACE.

Variables	AUC	95%CI	P	Cut-off	Sensitivity	Specificity
GLS	0.702	0.604 ~ 0.801	<0.001	11.45	0.390	0.716
MVO	0.637	0.530 ~ 0.744	0.009	1.91	0.585	0.707
hs-TnT	0.659	0.565 ~ 0.753	0.002	2450.5	0.780	0.509
Integral ECV	0.633	0.533 ~ 0.733	0.011	33.16	0.439	0.793
NMI-ECV	0.562	0.455 ~ 0.670	0.236	30.16	0.244	0.966
MI-ECV	0.639	0.537 ~ 0.741	0.008	48.18	0.610	0.655

GLS, global longitudinal strain; MVO, microvascular obstruction; hs-TnT, high sensitivity troponin T; MI, myocardial infarction; NMI, non-myocardial myocardial infarction.

## Discussion

To the best of our knowledge, there are currently no relevant data in the diabetes cohort to demonstrate the consistency and correlation between synthetic and conventional ECV. The prognostic value of different regional ECV in diabetes patients is unclear. The main findings of this study are as follows. First, Synthetic ECV was strongly consistent and correlated with conventional ECV in T2DM patients with AMI. Second, Elevated synthetic ECV was an independent risk factor for MACE in T2DM patients with AMI. Third, Patients with high synthetic ECV had a significantly higher long-term risk of MACE.

### ECV in T2DM patients with AMI

T2DM is a high-risk factor for MI and is associated with a high prevalence of diastolic dysfunction and congestive heart failure (28). A potential contributing factor is the accelerated accumulation of diffuse myocardial fibrosis and stiffness (29). Although biopsy remains the gold standard for assessing myocardial fibrosis, this invasive test is difficult to perform in the acute phase of AMI. ECV assessed by CMR is a marker of cardiac remodeling in the early stages of a variety of cardiac diseases and is associated with poor clinical outcomes (11, 30). Therefore, ECV may be of significant value in patients with AMI combined with diabetes. In a previous study of patients with T2DM, ECV levels were lower than in our study (27.9 ± 2.6% vs. 30.44 ± 6.5%) (11, 30). This may be because all patients included in our study combined with AMI, and acute myocardial injury may have additionally increased ECV levels.

### Establishment and validation of an ECV model without blood sampling

HCT is crucial for the calculation of conventional ECV. Due to the high temporal variability of HCT, it should be as close as possible to the CMR scan time (12–15). However, this is still not an immediate HCT. It has been reported that HCT levels may even change within a few hours (12). Thus, these factors limit the applicability of ECV in routine clinical work. Recently, synthetic ECV without blood sampling has received considerable attention. The calculation of synthetic ECV is based on a linear relationship between the native R1 (1/T1) and HCT (16–19). In contrast to conventional HCT, synthetic HCT is measured during CMR scanning and is calculated from the R1 of LV blood. Thus, it somewhat avoids potential variability caused by differences in LV and peripheral venous blood or changes in body position and time that may affect conventional HCT measurements (32–34). Although some synthetic ECV models have been previously established and validated (16, 20–23), no relevant reports have been seen for diabetic patients. In this study, we established and validated the first synthetic ECV model containing only T2DM patients with AMI. Similar to previous studies, synthetic HCT was only moderately correlated with conventional HCT (16, 20, 21, 35, 36). However, the performance of synthetic ECV was favorable. The close correlation between synthetic ECV and conventional ECV may be attributed to the four additional terms (myocardial and ventricular blood R1 before and after contrast injection) that remained constant in the ECV calculations, and these constants may have partially offset the greater variability in HCT. In contrast, the differences between HCT may be related to the following reasons. First, there is high variability in HCT itself as described above. Second, blood iron outside hemoglobin has been reported to have a substantial effect on T1 relaxation time, and the R1/HCT relationship may be broken in patients with iron overload, especially those with thalassemia (37). In addition, conventional HCT is measured in peripheral venous blood, whereas synthetic HCT is derived from the T1 relaxation time of left ventricular arterial blood. Differences between arterial and venous blood may also introduce interference. In our study, we successfully established and validated a synthetic ECV model in T2DM patients with AMI. Based on the unique value of ECV for myocardial fibrosis, the role of this “pragmatic” ECV in patient prognosis is equally attractive. However, given the characteristics of HCT, the synthesis of HCT and ECV needs to be repeatedly verified in different subpopulations.

### Prognostic value of synthetic ECV in T2DM patients with AMI

Elevated ECV is associated with poor prognosis in previous diabetes cohorts and animal studies (11, 38). In our study, it was found that elevated synthetic ECV was an independent risk factor for MACE in T2DM patients with AMI, patients with high synthetic ECV had a significantly higher long-term risk of MACE. Although the exact mechanism of action is unknown, myocardial fibers may be an important cause. Specifically, myocardial fibrosis is one of the core mechanisms of myocardial remodeling and poor prognosis. In diabetic patients, myocardial fibrosis can be promoted through various pathways such as inflammation and oxidative stress. ECV, as a marker of myocardial fibrosis, may account for the findings of this study (38, 39). In addition, we found that GLS was also an independent risk factor for MACE in patients with AMI, and Integral ECV, NMI-ECV, and NMI-ECV were all significantly correlated with GLS, which quantifies systolic function by measuring LV deformation and is independent of geometric factors (40, 41). Even in the presence of normal LVEF, ECV, and GLS can detect LV disease and stratify risk (40, 42). Indeed, correlations between ECV and GLS have been demonstrated in other diseases (43, 44). These may also partly explain the results of the present study. In a previous study that included 47 patients with T2DM, high HbA1c was shown to be associated with increased ECV (45). In contrast, in a larger study, the association between HbA1c and ECV was not confirmed (31), which is consistent with our findings. The different results may be related to several reasons. First, sample size limitations may have led to some bias. In addition, the study populations were different. All T2DM patients in our study were combined with AMI, and it is known that myocardial necrosis is also an important factor in myocardial fibrosis. In another cohort that includes non-diabetic patients with AMI, ECV was also proven to be a powerful predictor of heart failure and all - cause mortality (46). Given the crucial role of myocardial fibrosis, it appears that ECV is closely associated with prognosis in both diabetic and non - diabetic AMI patients.

### Role of NMI-ECV in T2DM patients with AMI

In recent years, myocardial fibrosis in non-infarcted regions has received increasing attention. In the early stages of AMI, myocardial necrosis and systemic inflammatory responses can cause collagen deposition and remodeling in non-infarcted regions (47, 48). In a mouse animal model, Tsuda et al. (49) found pathological evidence of myocardial fibrosis in the non-infarcted regions in the early stages of AMI. In clinical studies, it was also found that diffuse myocardial fibrosis could occur in non-infarcted myocardium in the early stages of AMI and that elevated NMI-ECV was associated with poor LV remodeling in the chronic phase (50, 51). Consistent with these findings, we found that elevated NMI-ECV was an independent risk factor for MACE in T2DM patients with AMI. However, it is noteworthy that in the ROC analysis, the results showed no statistical significance between NMI-ECV and MACE. Indeed, the prognosis value of myocardial fibrosis in the non-infarcted regions is inadequate and controversial for the prognosis of patients. In a previous study, Marijianowski et al. (52) found that myocardial fibrosis in non-infarct regions was not associated with LV remodeling after MI in patients with end-stage heart failure. Therefore, the prognostic value of NMI-ECV in T2DM patients with AMI may be worth further investigation.

### Clinical implications

One of the major advantages of synthetic ECV is that it provides CMR laboratories with the convenience of ECV measurement without the blood samples. In addition, this study emphasizes the prognostic importance of synthetic ECV (including Integral ECV, NMI-ECV, and MI-ECV) in T2DM patients with AMI. These findings increase the potential of ECV in routine clinical CMR, which could facilitate the widespread use of CMR-ECV. Certainly, there are some potential barriers to implementing synthetic ECV in routine clinical practice. For instance, differences in CMR protocols among various institutions, cost concerns, or a lack of technical expertise.

### Limitations

First, the single-center, retrospective design limits generalizability. There is a potential selection bias due to exclusion of patients with poor imaging or severe comorbidities. Second, in our study, there is a lack of histopathological validation for ECV, especially considering the known heterogeneity of myocardial fibrosis in diabetic patients. Third, the sample size of this study is limited, and the follow-up time is relatively short. Longer-term data will strengthen the claims regarding prognosis. Fourth, there is high variability in HCT, so further studies after matching for age, BMI, and gender are expected. Fifth, our study included patients with AMI combined with T2DM, so some of the results may need to be replicated and validated in other diseases.

## Conclusions

Synthetic ECV is strongly consistent and correlated with conventional ECV in T2DM patients with AMI. Elevated synthetic ECV is an independent risk factor for MACE in T2DM patients with AMI.

## Data Availability

The raw data supporting the conclusions of this article will be made available by the authors, without undue reservation.

## Supplementary material

Supplementary Figure 1 Workflow.

Supplementary Figure 2 Measurement of ECV. **(A)** Native T1 map; **(B)** Enhanced T1 map; **(C)** Generate ECV images; **(D)** The distribution coefficient λ generates the image; Red circle marks the endocardium, green circle marks the epicardium, ROI (yellow) marks the blood pool, ROI (pink) marks the non-myocardial infarction sites, ROI (blue) marks the myocardial infarction site.

Supplementary Figure 3 Linear regression analysis between hematocrit (HCT) and blood pool T1.

Supplementary Figure 4 Pearson analysis between synthetic ECV and HbA1c. NMI, non-myocardial infarction regions; MI, myocardial infarction regions; ECV, extracellular volume.
