# Supplementary figures and images for: Establishment of a synthetic ECV model and its prognostic value in diabetes patients with acute myocardial infarction
Source: Front Endocrinol (Lausanne). 2025 Jun 25;16:1534236. doi: 10.3389/fendo.2025.1534236 (PMC12237642; doi:10.3389/fendo.2025.1534236)

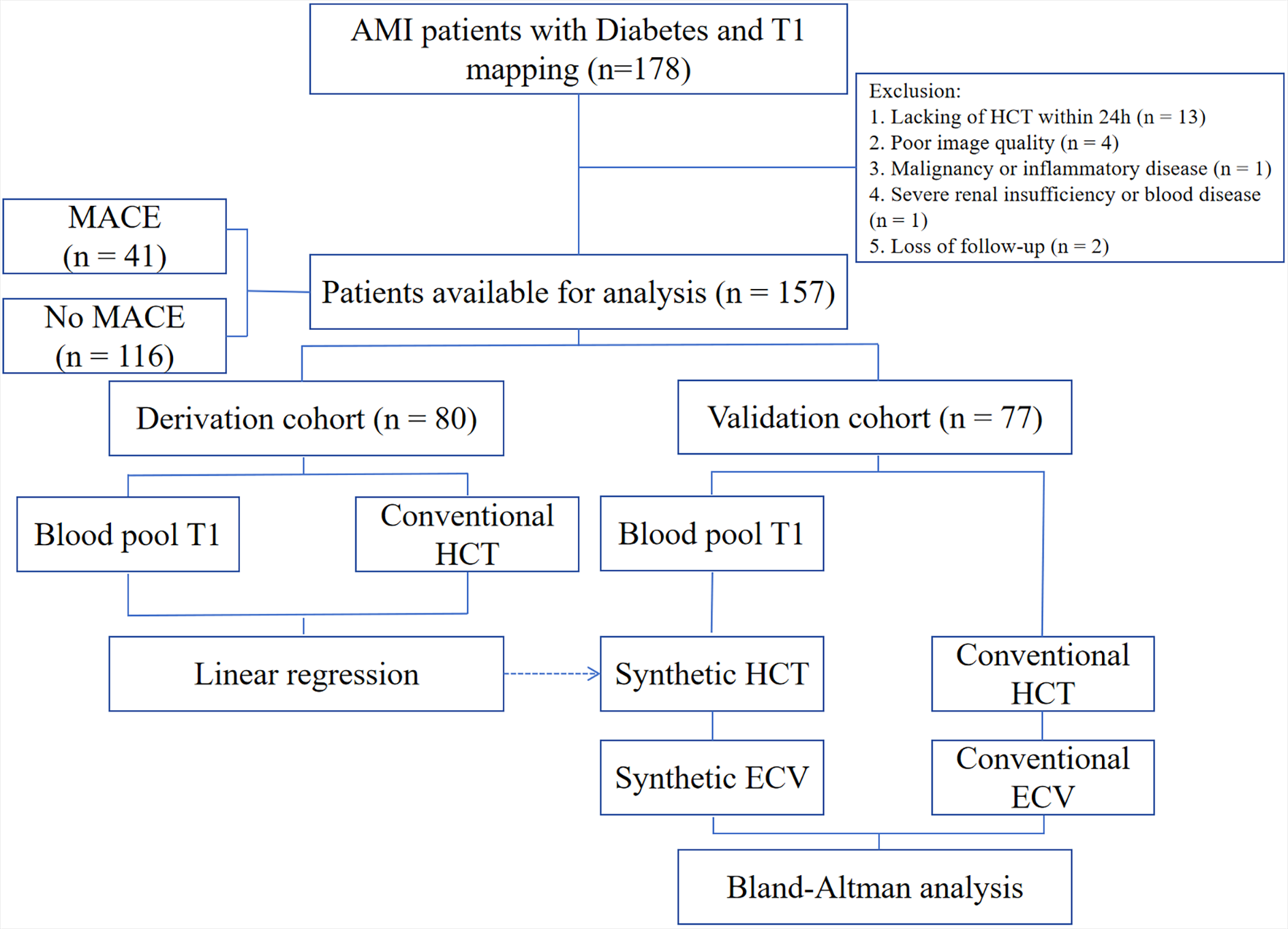

Supplement: Supplementary Figure 1 — Workflow. [file Image1.tif]

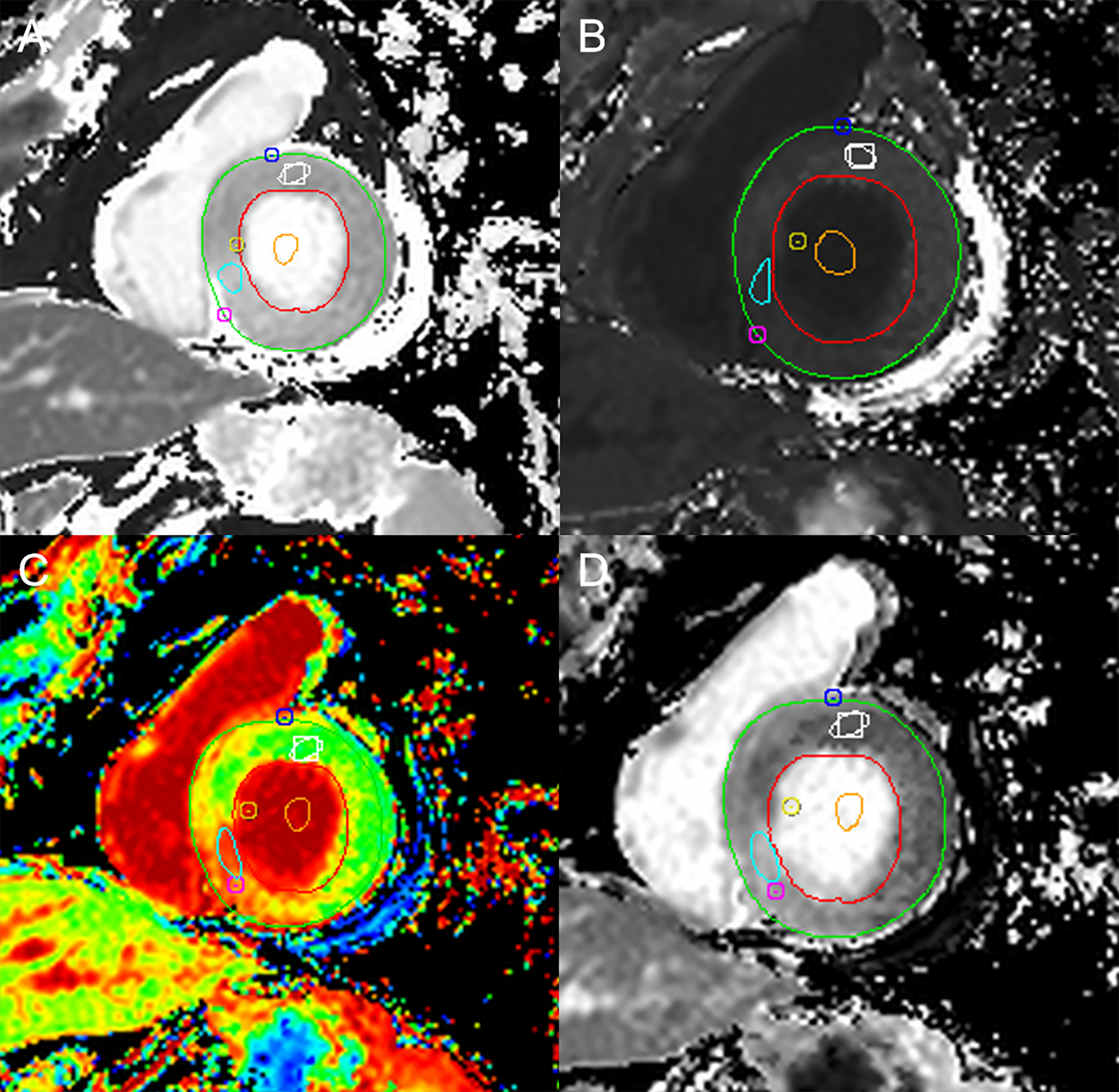

Supplement: Supplementary Figure 2 — Measurement of ECV. (A) Native T1 map; (B) Enhanced T1 map; (C) Generate ECV images; (D) The distribution coefficient λ generates the image; Red circle marks the endocardium, green circle marks the epicardium, ROI (yellow) marks the blood pool, ROI (pink) marks the non-myocardial infarction sites, ROI (blue) marks the myocardial infarction site. [file Image2.tif]

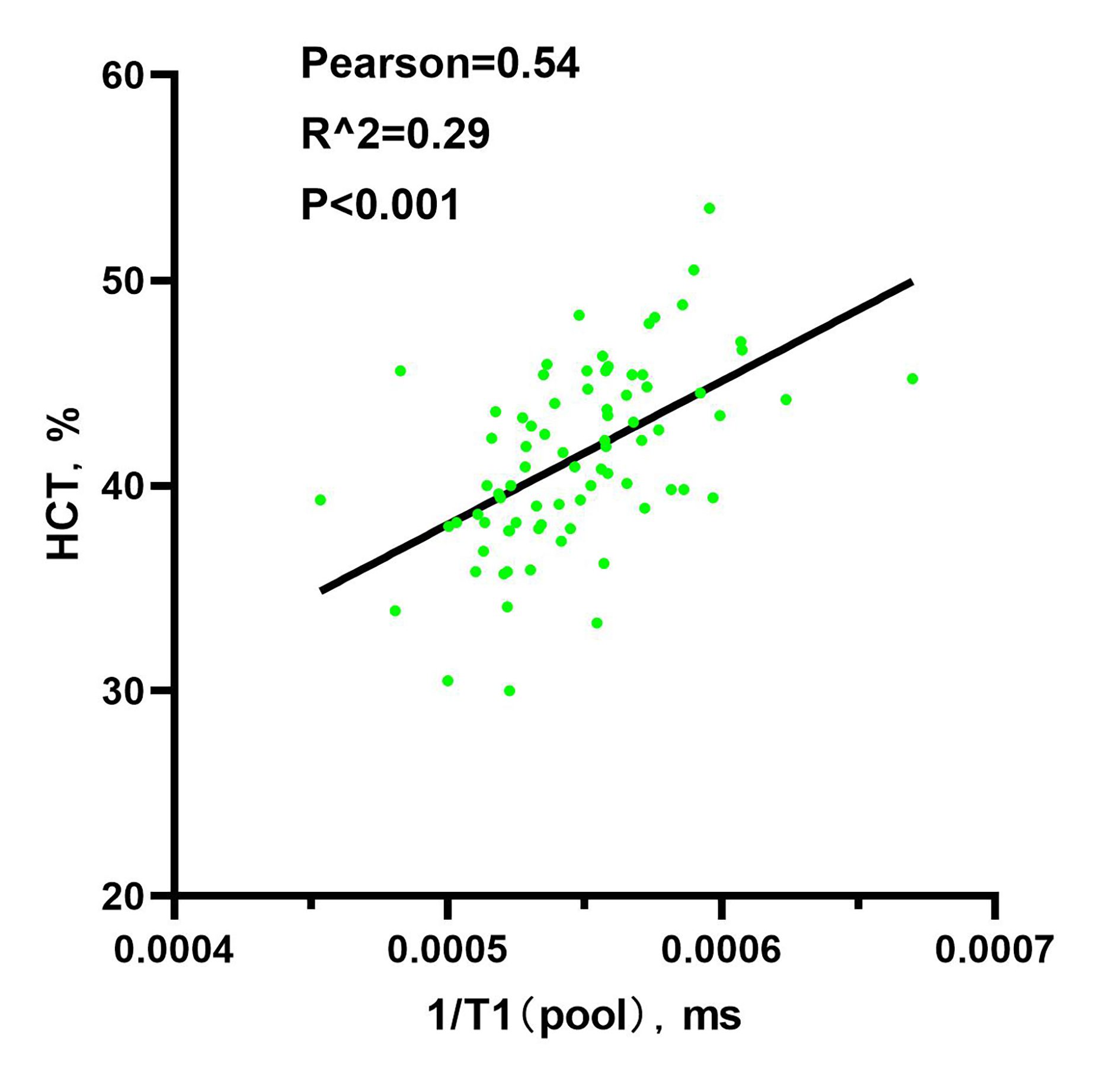

Supplement: Supplementary Figure 3 — Linear regression analysis between hematocrit (HCT) and blood pool T1. [file Image3.tif]

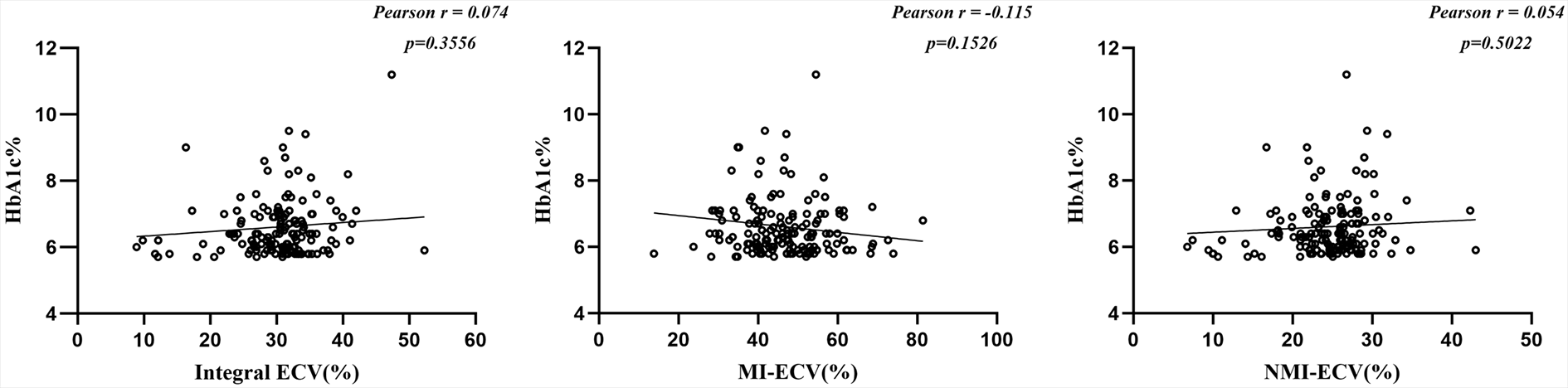

Supplement: Supplementary Figure 4 — Pearson analysis between synthetic ECV and HbA1c. NMI, non-myocardial infarction regions; MI, myocardial infarction regions; ECV, extracellular volume. [file Image4.tif]
