# Supplementary material for: Establishment of a synthetic ECV model and its prognostic value in diabetes patients with acute myocardial infarction
Source: Front Endocrinol (Lausanne). 2025 Jun 25;16:1534236. doi: 10.3389/fendo.2025.1534236 (PMC12237642; doi:10.3389/fendo.2025.1534236)
Supplement: Supplementary file 5 [file Table1.docx]

**Table S1** Patient characteristics

|  | Validation (n = 77) | Derivation (n = 80) | P |
| --- | --- | --- | --- |
| Age, years | 56.53 ± 10.96 | 59.48 ± 11.84 | 0.108 |
| Female, n (%) | 17 (22.08) | 10 (12.50) | 0.112 |
| BSA, m^2^ | 1.85 ± 0.17 | 1.81 ± 0.16 | 0.115 |
| BMI, kg/m^2^ | 26.07 ± 3.34 | 25.62 ± 3.11 | 0.387 |
| Current smoker, n (%) | 37(48.1) | 35 (48.3) | 0.589 |
| Hypertension, n (%) | 37 (48.05) | 32 (40.00) | 0.310 |
| Stroke, n (%) | 7(9.1) | 13 (16.3) | 0.179 |
| MVO, n (%) | 38(49.4) | 51 (63.7) | 0.069 |
| STEMI, n (%) | 48 (62.3) | 54 (67.5) | 0.498 |
| SBP, mmHg | 131.21±18.74 | 127.79±19.4 | 0.263 |
| DBP, mmHg | 78.58±12.02 | 80.64±14.74 | 0.341 |
| Heart rate, bpm | 79.6±13.06 | 80.8±16.12 | 0.609 |
| Total cholesterol, mmol/L | 4.38±1.15 | 4.29±0.89 | 0.619 |
| Triglycerides, mmol/L | 1.99±1.48 | 1.83±1.47 | 0.479 |
| HDL cholesterol, mmol/L | 0.96±0.22 | 0.95±0.23 | 0.802 |
| LDL cholesterol, mmol/L | 2.82±0.99 | 2.58±0.83 | 0.105 |
| HbA1c, % | 6.6±1.35 | 6.61±0.95 | 0.968 |
| FBG, mmol/L) | 8.83±3.28 | 9±3.49 | 0.750 |
| eGFR, mL/min/1.73 m2 | 110.66±16.09 | 107.20±17.19 | 0.196 |
| hs-TnT, ng/L | 2501.00 (1027.00, 3596.00) | 4669.00 (682.00, 6547.50) | 0.113 |
| NT-proBNP, pg/mL | 931.00 (587.90, 1544.00) | 1517.00 (561.00, 2625.00) | 0.100 |
| Aspirin, n (%) | 76 (98.70) | 77 (96.25) | 0.640 |
| P2Y12 inhibitors, n (%) | 65 (84.42) | 72 (90.00) | 0.294 |
| Statins, n (%) | 75 (97.40) | 75 (93.75) | 0.470 |
| Dapagliflozin, n (%) | 58 (75.32) | 57 (71.25) | 0.564 |
| ACEI/ARB, n (%) | 53 (68.83) | 48 (60.00) | 0.248 |
| β-Blockers, n (%) | 66 (85.71) | 69 (86.25) | 0.923 |
| Spironolactone, n (%) | 8 (10.39) | 10 (12.50) | 0.678 |
| Killip class≥2, n (%) | 6 (7.8) | 9 (11.3) | 0.461 |
| IRA-LCX, n (%) | 19(24.7) | 17 (21.3) | 0.610 |
| IRA-LAD, n (%) | 28 (36.4) | 37 (46.3) | 0.209 |
| IRA-RCA, n (%) | 29 (37.7) | 24 (30.0) | 0.310 |
| Integral ECV, % | 29.53 ± 5.50 | 31.01 ± 6.94 | 0.142 |
| NMI-ECV, % | 24.31 ± 5.07 | 24.49 ± 5.81 | 0.842 |
| MI-ECV, % | 46.45 ± 10.76 | 46.97 ± 10.35 | 0.760 |
| GLS, % | 13.42±4.11 | 12.69±4.66 | 0.297 |
| LVEF, % | 46.66 ± 10.25 | 44.36 ± 11.33 | 0.184 |
| LV-EDVi, mL/m^2^ | 75.55±17.05 | 79.75±21.72 | 0.181 |
| LV-ESVi, mL/m^2^ | 43.00±15.87 | 46.71±18.53 | 0.180 |
| MVO% | 22.16 (15.38, 31.72) | 27.08 (17.70, 39.86) | 0.074 |
| LGE% | 0 (0, 3.66) | 1.18 (0, 4.79) | 0.078 |
| Native myo T1-time, ms | 1342.25 ± 81.32 | 1361.33 ± 75.08 | 0.128 |
| Native LV blood T1-time, ms | 1792.09 ± 142.91 | 1822.21 ± 108.66 | 0.138 |
| HCT, % | 42.03 ± 4.22 | 41.65 ± 4.57 | 0.589 |

BMI = body mass index; STEMI, ST-segment elevation myocardial infarction; GFR = glomerular filtration rate; ECV = extracellular volume; LVEF = left ventricular ejection fraction; GLS = global longitudinal strain; LV = left ventricular; EDVi = end-diastolic volume index; LGE = late gadolinium enhanced; MVO = microvascular obstruction; HCT = haematocrit; ESVi = end-systolic volume index; SBP = systolic blood pressure; DBP = diastolic blood pressure; LAD = left atrial diameter; LCX = left circumﬂex artery; RCA = right coronary artery; ACEI = angiotensin-converting-enzyme inhibitor; IRA = infarct-related artery; ARB = angiotensin II receptor blocker; HDL-C = high-density leptin cholesterol; LDL-C = low-density leptin cholesterol; Others = left main coronary artery and intermediate branch; hs-CRP = high sensitivity C-reactive protein; hs-TnT = high sensitivity troponin T; NT-proBNP = N-terminal pro-B-type natriuretic peptide; MI = myocardial infarction; NMI = non-myocardial infarction.
