# Supplementary material for: Establishment of a synthetic ECV model and its prognostic value in diabetes patients with acute myocardial infarction
Source: Front Endocrinol (Lausanne). 2025 Jun 25;16:1534236. doi: 10.3389/fendo.2025.1534236 (PMC12237642; doi:10.3389/fendo.2025.1534236)
Supplement: Supplementary file 6 [file Table2.docx]

| **Adverse events, n (%)** | **All patients (n = 157)** |
| --- | --- |
| MACE | 41 (26.1) |
| All-cause death | 3 (1.9) |
| Recurrent myocardial infarction | 7 (4.5) |
| Stroke | 4 (2.5) |
| Heart Failure | 27 (17.2) |

**Table S2** Adverse Events During Follow-up
